# Intimate partner violence and its association with mental health problems: The importance of childhood violence – The SAMINOR 2 Questionnaire Survey

**DOI:** 10.1177/14034948211024481

**Published:** 2021-06-30

**Authors:** Astrid M. A. Eriksen, Marita Melhus, Bjarne K. Jacobsen, Berit Schei, Ann-Ragnhild Broderstad

**Affiliations:** 1 Centre for Sami Health Research, UiT The Arctic University of Norway, Norway; 2 Department of Community Medicine, UiT The Arctic University of Norway, Norway; 3 Department of Public Health, NTNU, Norway; 4 Department of Obstetrics and Gynecology, St. Olav`s Hospital, Trondheim University Hospital, Norway

**Keywords:** Intimate partner violence, childhood violence, Sami, mental health, post-traumatic stress, HSCL-10, psychological distress, emotional violence, physical violence, sexual violence

## Abstract

**Aims:**

This study aims to estimate the prevalence of intimate partner violence (IPV) and its association with psychological distress and symptoms of post-traumatic stress (PTS) among Sami and non-Sami and to explore whether the association between IPV and mental health is modified by exposure to childhood violence (CV). These issues are scarcely studied among the Sami.

**Methods:**

This study was based on the cross-sectional SAMINOR 2 Questionnaire Survey, a part of the Population-based Study on Health and Living Conditions in Regions with Sami and Norwegian Populations (SAMINOR). Chi-square tests and two-sample *t*-tests were used to test differences between groups. Multiple linear regression analysis was applied to explore the association between IPV/CV and continuous scores of psychological distress and symptoms of post-traumatic stress.

**Results:**

Experiences of IPV (emotional, physical, and/or sexual) were reported by 12.8% of women and 2.0% of men. A significantly higher proportion of Sami women reported exposure to emotional (12.4 v. 9.5%, *p* = 0.003), physical (11.6 v. 6.9%, *p* < 0.001), and any IPV (17.2 v. 11.8%, *p* < 0.001) compared to non-Sami women. There were no ethnic differences in sexual IPV among women (2%). Exposure to IPV was associated with a higher score of psychological distress and PTS and was highest among those exposed to both IPV and CV.

**Conclusions:**

Sami women reported the highest prevalence of IPV. The association between IPV/CV and mental health problems did not differ by ethnicity or gender. The most severe mental health problems were observed for those who were exposed to both IPV and CV.

## Background

According to the World Health Organization (WHO), intimate partner violence (IPV) includes physical, sexual and emotional abuse and controlling behaviour by an intimate partner and is a serious public health issue that adversely affects both mental and physical health [1]. The magnitude and pattern of IPV vary across countries, regions, genders and ages [2,3]. Even though some studies have found that men report being victims of violence just as often as women, women are far more likely than men to be injured during assaults by intimate partners, and women suffer from both sexual and more severe forms of violence [1]. The WHO has identified IPV as the most common form of violence against women [4]. It is estimated that approximately 30% of women who have been in relationships have experienced physical and/or sexual violence at the hands of an intimate partner [1]. Broad ranges of health outcomes, including mental health problems, have been associated with IPV among women [5,6]. Mental health problems are also strongly associated with a history of child abuse [7,8]. Furthermore, abused children are at high risk of further victimisation as adolescents or adults [9]. Given that many of those who experience childhood violence (CV) are also exposed to IPV, it is important to investigate the impact of experiencing both [10,11].

In Norway, a country with a comprehensive welfare system and high gender equality, the first population-based study on IPV among women and men was conducted in 2003/2004 and demonstrated an extensive use of physical power and violence in intimate relationships and a higher prevalence of IPV in women than in men [12]. Another national population-based survey conducted in 2013 revealed that women reported a higher prevalence of IPV and also a higher number of violent episodes than men did [13]. A total of 41% of women exposed to rape reported that the perpetrator was a partner/former partner; the corresponding figure among men was 13%. None of these studies measured the exposure to emotional/psychological IPV.

Globally, studies on IPV among indigenous populations are sparse. A systematic review from 2017 identified 13 studies that focused on indigenous populations, IPV and mental health [14]. Most of these studies were conducted in North America (nine studies), followed by Australia, New Zealand, the Pacific Islands and Asia (one study each). Most studies reported high rates of IPV, although methodological difficulties were identified. The most commonly reported mental health outcomes were depression and post-traumatic stress disorder (PTSD) [14]. Studies have shown that indigenous women from Canada are two to three times more likely than non-indigenous women to report IPV from a current or former partner [15,16].

The Sami are an ethnic minority living in Norway, Sweden, Finland and the Kola Peninsula of the Russian Federation. Most of the Sami live in Norway, where they are recognised as the indigenous people. The Sami have been subjected to harsh assimilation policies for over 100 years. Health disparities between indigenous peoples and their majority populations have often been linked to colonisation, forced assimilation, violence and discrimination [16,17]. A number of media articles have focused on sexual violence in Sami communities, but little research on IPV and the association between IPV and mental health has been done among the Sami [18,19]. A previous study revealed that Sami participants reported more violence in both childhood and adulthood compared to non-Sami participants [20]. However, that study addressed violence in general and did not focus on the relationship between the victim and the abuser. Another study found an association between childhood abuse and mental health symptoms in adulthood [21]. To our knowledge, there has not been any population-based study on IPV alone or IPV in combination with CV in the Sami compared to the non-Sami population in Norway.

### Aims of the study

The aims of the study were (a) to estimate the prevalence of IPV among Sami and non-Sami women and men; (b) to investigate the association between IPV and mental health problems (psychological distress and symptoms of PTS) and explore whether these associations differed between Sami and non-Sami; and (c) to determine whether the association between IPV and mental health problems was modified by exposure to CV.

## Materials and methods

This study was based on the cross-sectional SAMINOR 2 Questionnaire Survey, part of the second wave of the Population-based Study on Health and Living Conditions in Regions with Sami and Norwegian Populations (the SAMINOR Study) [22]. The survey was conducted in 2012 by the Centre for Sami Health Research, UiT The Arctic University of Norway. The questionnaire, including an English translation, is available at www.saminor.no.

### Sample

The study population included all inhabitants aged 18–69 years living in 25 selected multi-ethnic municipalities (mixed Sami and non-Sami populations) in Northern and Central Norway (in six of the municipalities, only selected districts were included). Out of 43,245 invitees, 11,600 participated, yielding a participation rate of 27%. In the present study, 96 respondents were excluded due to missing information on ethnicity, 121 due to missing information concerning violence, 567 due to missing information on three or more items on the 10-item version of the Hopkins Symptoms Checklist (HSCL-10), and 26 due to missing information on all three questions regarding symptoms of PTS. Thus, the total analytic sample consisted of 10,790 participants: 6003 (55.6%) women and 4787 (44.4%) men.

### Ethnicity

Ethnicity was categorised as Sami or non-Sami based on information collected from the questionnaire. To be categorised as Sami, two criteria had to be met: the subjective criterion of self-perceived Sami ethnicity (whether the respondents considered themselves to be Sami) and the more objective criterion of Sami linguistic affiliation (if at least one grandparent, parent, or the participants themselves use(d) Sami as their household language). These criteria resemble those used by the Norwegian Sami Parliament to register voters. All other participants were categorised as non-Sami. Of the 10,790 participants, the use of this classification resulted in 2116 Sami (19.6%) and 8674 non-Sami (80.4%). However, as there is no consensus on how to define Sami ethnicity, and different classifications have been in operation [23,24], sensitivity analyses were performed using an alternative ethnic categorisation. In this alternative, the subjective criterion was changed to a positive answer to at least one of the following questions: ‘I consider myself Sami’ and ‘My ethnic background is Sami’. The same objective criterion of Sami linguistic affiliation was also applied, and participants who met both of these criteria were classified as Sami and all others, as non-Sami. This alternative ethnic categorisation expanded the Sami group to 2603 (1141 (23.8%) Sami men and 1462 (24.4%) Sami women).

### IPV and childhood violence

Experience with emotional, physical and sexual violence was measured using questions from the Norvold Abuse Questionnaire (NorAQ), with one question for each type of violence. In their responses, participants could indicate whether the violence occurred in adulthood and/or in childhood and could indicate the perpetrator with the following response options: ‘Stranger’, ‘Spouse’ (married or cohabiting partner), ‘Family/relative’ and/or ‘Other acquaintance’. Multiple answers were allowed.

Participants who answered ‘Yes, as an adult’ and/or ‘Yes, past 12 months’ to the question, ‘Has anyone ever systematically and over a long period tried to subdue, degrade, or humiliate you?’ and in addition, ticked ‘Spouse/partner’ as the perpetrator, were classified as exposed to emotional IPV. The remaining respondents were classified as non-exposed. Participants who answered ‘Yes, as an adult’ and/or ‘Yes, past 12 months’ to the question, *‘*Have you experienced physical attacks/abuse?’ and ticked ‘Spouse/partner’ as the perpetrator, were classified as exposed to physical IPV, and the remaining respondents were classified as non-exposed. Participants who answered ‘Yes, as an adult’ and/or ‘Yes, past 12 months’ to the question, ‘Have you been sexually abused?’ and in addition, ticked ‘Spouse/partner’ as the perpetrator, were classified as exposed to sexual IPV, and the remaining respondents were classified as non-exposed. Participants categorised as exposed to one or more of the aforementioned types of IPV (emotional, physical, sexual) were also pooled and further categorised as exposed to any IPV, with the remaining respondents classified as non-exposed.

Participants who answered ‘Yes, as a child’ to at least one of the questions ‘Has anyone ever systematically and over a long period tried to subdue, degrade, or humiliate you?’, *‘*Have you experienced physical attacks/abuse?’, and ‘Have you been sexually abused?’ were classified as exposed to any CV, and the remaining respondents were classified as non-exposed.

A four-category, combined IPV/CV variable was then constructed for each type of IPV:

1. ‘No emotional/physical/sexual/any IPV and no CV’;
2. ‘Any CV, but no emotional/physical/sexual/any IPV’;
3. ‘Emotional/physical/sexual/any IPV, but no CV’;
4. ‘Emotional/physical/sexual/any IPV, and any CV’.

The category ‘No emotional/physical/sexual/any IPV and no CV’ was used as the reference category. However, as a person who has never experienced, for instance, sexual IPV, may have experienced other types of IPV or violence in adulthood from a person other than their partner, the reference group ‘No sexual IPV and no CV’ may include participants who have experienced other types of violence. Sensitivity analyses were therefore performed using only participants who reported no violent experiences in their lifetime as the reference group.

### Mental health problems

Psychological distress was measured using the HSCL-10, which is primarily comprised of symptoms of anxiety and depression [25]. The HSCL-10 addresses respondents’ experiences of (1) sudden anxiety, (2) anxiousness, (3) dizziness, (4) tension/stress, (5) self-blame, (6) sleeplessness, (7) sadness, (8) worthlessness, (9) finding everything burdensome and (10) hopelessness during the four previous weeks. Each item was rated on a 4-point scale, from 1 ‘Not at all bothered’ to 4 ‘Extremely bothered’. For respondents with one or two missing items, missing values were replaced with the sample mean value for each item, as suggested by Strand et al. [25]. There were 241 (2.2%) participants with one missing item and 44 (0.4%) with two missing items. A psychological distress score was then calculated as the mean of the 10 items in HSCL-10, producing a score ranging from 1 to 4, where 1 indicated no symptoms of anxiety and depression, and 4 indicated severe symptoms. In the final sample, the internal consistency of the psychological distress score was high (Cronbach α = 0.90), with no ethnic differences. The score was used as a continuous variable in our study.

Symptoms of PTS during the last 12 months were assessed by posing three questions on (a) intrusive memories, (b) avoidance of certain situations, and (c) emotional numbness. The four response options (‘No’, ‘Yes, but rarely’, ‘Sometimes’ and ‘Often’) were ranked on a 4-point scale, from 1 ‘No’ to 4 ‘Often’. There were 56 (0.5%) participants with missing values on intrusive memories, 77 (0.7%) with missing values on avoidance of certain situations and 105 (1.0%) with missing values on emotional numbness. Missing values were recoded to 1 ‘No’. A PTS score was calculated as the mean of the three items and used as a continuous variable in the analyses.

### Background variables

#### Laestadian affiliation

Respondents were asked to indicate their affiliation with any of the following religious/life-stance organisations: ‘the state church’ (Church of Norway), ‘the Laestadian congregation’, ‘other religious organisation/community’, ‘non-religious life-stance organisation/community’, ‘not a member of any religious/life-stance organisation’. Multiple answers were allowed. Participants who reported that they themselves, their mother, father, or grandparents were affiliated with a Laestadian congregation were classified as Laestadianists. The Laestadian movement is a conservative Lutheran denomination particularly widespread among the Sami in the northern regions of Norway, Sweden and Finland. Respondents with no personal or familial affiliation to a Laestadian congregation were classified as non-Laestadianists (missing *n* = 145, 1.3%). The vast majority of participants were affiliated with the state church.

#### Municipality of residence

The 25 municipalities included in the SAMINOR 2 Questionnaire Survey were selected based on the 1970 census in Norway and other relevant knowledge indicating a significant Sami population [26]. However, the density of Sami in these municipalities differs. Five municipalities in the former Finnmark County (Kautokeino, Karasjok, Porsanger, Tana and Nesseby) were defined as Sami majority regions. In these municipalities or in certain areas of the municipality, a majority of the population are of Sami descent, and Sami culture and language are dominant [26]. Regions in which Sami are considered a minority were categorised as Sami minority regions and included the remaining municipalities: Røyrvik, Snåsa, Røros, Namsskogan, Narvik, Grane, Hattfjelldal, Tysfjord, Evenes, Skånland, Lavangen, Lyngen, Storfjord, Kåfjord, Kvænangen, Alta, Loppa, Kvalsund, Lebesby and Sør-Varanger.

#### Exposure to discrimination

Respondents were asked about their exposure to discrimination with the following question: ‘Have you been subjected to discrimination?’ The response options were: ‘Yes, during the last two years’, ‘Yes, previously’, ‘No’, and ‘Don’t know’. The two positive response options were merged. There were 63 missing values (0.6%), and they were included in the ‘No’ group.

#### Alcohol intake

Respondents were asked to indicate how often they had consumed alcohol in the last year. The eight possible response options were collapsed into three categories: Seldom/never (including the responses ‘Never consumed alcohol’, ‘Have not been drinking alcohol during the last year’, ‘A few times during the last year’), Monthly (‘About once a month’, ‘Two or three times per month’), and Weekly (‘About once a week’, ‘Two or three times a week’, ‘Four to seven times a week’). There were 79 missing values for alcohol intake (0.7%).

#### Sociodemographic variables

Age and gender were retrieved from the National Population Registry. Duration of education was used as a proxy for socioeconomic status and was reported in the questionnaire as the completed number of years of education. Age and duration of education were used as continuous variables in multivariable regressions. There were 93 participants with missing values for duration of education (0.9%).

### Ethics

The data collection and storage of data were approved by the Norwegian Data Protection Authority (Datatilsynet). Written informed consent was obtained from all participants. The study was approved by the Regional Committee for Medical and Health Research Ethics (REK-Sør-Øst) and the SAMINOR Project Board.

### Statistical analysis

IBM SPSS Statistics Version 26.0 for Windows was used to conduct statistical analyses. Categorical sample characteristics are presented as numbers and percentages, and continuous variables are presented as means and standard deviations, stratified by gender and ethnicity. The Pearson’s chi-square test was used for categorical variables, and the two-sample *t*-test was used for continuous variables for the comparison between Sami and non-Sami. For the continuous psychological distress and PTS scores, means, standard deviations, medians and first and third quartiles are presented. The comparison of means of psychological distress and PTS scores between the two ethnicities was performed using two-sample *t*-tests. A non-parametric test (Wilcoxon rank-sum test) was also performed. Multiple linear regression analyses were conducted with psychological distress score and PTS score as dependent variables, and the four-category, combined IPV/CV variables were used as main predictors in separate models. Separate models were run for Sami, non-Sami and all women, and for emotional, physical, sexual and any IPV. Due to the low number of men exposed to IPV, only an analysis for any IPV was performed for all men combined. Results are presented as beta coefficients and *p*-values. In each case, two models were run; in model 1, age was included as a possible confounder. In model 2, age, duration of education, Laestadian affiliation, area of residence (Sami majority/Sami minority), exposure to discrimination and alcohol intake were included as possible confounders. We investigated a possible interaction between ethnicity and the four-category, combined IPV/CV variables on the two dependent variables reflecting mental health problems. Level of significance was set at 5%.

## Results

Background characteristics of the study sample are presented in Table I. Sami women were younger than the non-Sami women (mean age 44.9 and 46.3 years, respectively, *p* < 0.001), whereas there were no significant ethnic differences in age among men (mean age 49.3). The mean duration of education was 14.6 years for Sami women v. 13.9 for non-Sami women (*p* < 0.001). The corresponding figures for men were 13.0 for both ethnicities (*p* = 0.607). Laestadian affiliation was two and a half times more common among Sami compared to non-Sami. A higher proportion of Sami reported exposure to discrimination compared to non-Sami. Moreover, Sami reported less frequent alcohol intake than non-Sami (Table I).

**Table I.** Background characteristics of the study sample by gender and ethnicity: the SAMINOR 2 Questionnaire Survey (*n* = 10,790).

	Women (*n* = 6003)					Men (*n* = 4787)				
	Sami (*n* = 1197)		non-Sami (*n* = 4806)			Sami (*n* = 921)		non-Sami (*n* = 3866)		
	Mean	SD	Mean	SD	*p*-value^a^	Mean	SD	Mean	SD	*p*-value^a^
Age (years)	44.9	13.9	46.3	13.5	0.001	49.0	13.4	49.4	13.3	0.500
Duration of education (years)	14.6	3.9	13.9	3.7	< 0.001	13.0	3.8	13.0	3.7	0.607
	*n*	%	*n*	%	*p*-value^a^	*n*	%	*n*	%	*p*-value^a^
Laestadianism affiliation					< 0.001					< 0.001
Non-Laestadianist	699	54.4	4015	83.5		535	58.1	3241	83.8	
Laestadianist	498	41.6	791	16.5		386	41.9	625	16.2	
Municipality of residence					< 0.001					< 0.001
Sami majority region	740	61.9	539	11.2		551	59.8	417	10.8	
Sami minority region	457	38.1	4267	88.8		370	40.2	3447	89.2	
Exposed to discrimination					< 0.001					< 0.001
No	566	47.3	3855	80.2		444	48.2	3136	81.1	
Yes	505	42.2	633	13.2		372	40.4	454	11.7	
Don’t know	126	10.5	318	6.6		105	11.4	276	7.1	
Alcohol intake					< 0.001					0.007
Seldom/never	520	43.2	1755	36.5		261	28.3	1025	26.5	
Monthly	451	37.7	1721	35.8		367	39.8	1393	36.0	
Weekly	226	18.9	1330	27.7		293	31.8	1448	37.5	

a Comparing Sami and non-Sami by Pearson’s chi-squared test.

### Intimate partner violence and childhood violence

A total of 12.8% of women (Table II) and 2.0% of men (results not shown in table) reported any IPV. A significantly higher proportion of Sami women reported emotional (12.4 v. 9.5%, *p* = 0.003), physical (11.6 v. 6.9%, *p* < 0.001), and any IPV (17.2 v. 11.8%, *p* < 0.001) compared to non-Sami women (Table II). There were no ethnic differences in the reporting of sexual IPV among women (2.1 v. 1.8%, *p* = 0.524) (Table II). Any CV was more commonly reported by Sami than by non-Sami women (31.2 v. 21.6%, *p* < 0.001) (Table II). Further, exposure to IPV was strongly associated with exposure to CV. Among women who reported any CV, a total of 19.6% reported IPV, while 10.8% of women who did not report CV reported IPV (results not shown in table). The same pattern was found among men; 2.5% of men exposed to CV reported any IPV as compared with 1.5% of men not exposed to CV (results not shown in the table).

**Table II.** Prevalence of intimate partner violence (IPV) and childhood violence (CV) among all women and by ethnicity, the SAMINOR 2 Questionnaire Survey (*n* = 6003).

Violence	All women (*n* = 6003)		Sami women (*n* = 1197)		Non-Sami women (*n* = 4806)		*p*-value^a^
	*n*	%	*n*	%	*n*	%	
Any CV							< 0.001
No	4591	76.5	823	68.8	3768	78.4	
Yes	1412	23.5	374	31.2	1038	21.6	
Any IPV							< 0.001
No	5232	87.2	991	82.8	4241	88.2	
Yes	771	12.8	206	17.2	565	11.8	
Emotional IPV (Yes)	603	10.0	148	12.4	455	9.5	0.003
Physical IPV (Yes)	470	7.8	139	11.6	331	6.9	< 0.001
Sexual IPV (Yes)	112	1.9	25	2.1	87	1.8	0.524
Emotional IPV and/or any CV							< 0.001
No emotional IPV and no CV	4203	70.0	737	61.6	3466	72.1	
Any CV, but no emotional IPV	1197	19.9	312	26.1	885	18.4	
Emotional IPV, but no CV	388	6.5	86	7.2	302	6.3	
Emotional IPV and any CV	215	3.6	62	5.2	153	3.2	
Physical IPV and/or any CV							< 0.001
No physical IPV and no CV	4285	71.4	745	62.2	3540	73.7	
Any CV, but no physical IPV	1248	20.8	313	26.1	935	19.5	
Physical IPV, but no CV	306	5.1	78	6.5	228	4.7	
Physical IPV and any CV	164	2.7	61	5.1	103	2.1	
Sexual IPV and/or any CV							< 0.001
No sexual IPV and no CV	4514	75.2	808	61.7	3706	77.1	
Any CV, but no sexual IPV	1377	22.9	364	30.4	1013	21.1	
Sexual IPV, but no CV	77	1.3	15	1.3	62	1.3	
Sexual IPV and any CV	35	0.6	10	0.8	25	0.5	
Any IPV and/or any CV							< 0.001
No IPV and no CV	4097	68.2	704	58.8	3393	70.6	
Any CV, but no IPV	1135	18.9	287	24.0	848	17.6	
Any IPV, but no CV	494	8.2	119	9.9	375	7.8	
Any IPV and any CV	277	4.6	87	7.3	190	4.0	

a Comparing Sami and non-Sami by Pearson’s chi-squared test.

Among all women in the study sample, 4.6% reported both any IPV and any CV (Table II), while the corresponding figure for men was 0.5% (results not shown in the table). A higher proportion of Sami women reported both emotional IPV and any CV (5.2 v. 3.2%, *p* = 0.001), both physical IPV and any CV (5.1 v. 2.1%, *p* < 0.001), and both any IPV and any CV (7.3 v. 4.0%, *p* < 0.001) compared to non-Sami women (Table II). Very few women (< 1%) reported both sexual IPV and any CV, with no ethnic differences (*p* = 0.20) (Table II).

### Mental health problems

The mean psychological distress score was slightly higher among Sami women (1.40) compared to non-Sami women (1.37) (*p* = 0.034) (Table III). However, the distribution was skewed, and the median was found to be 1.20 for both ethnicities. Despite the equal median, the distributions were significantly different, with a higher portion of Sami in the upper part of the scale (*p* < 0.001 in Wilcoxon rank-sum test). The same pattern was observed for PTS score, with a mean of 1.61 and 1.49 for Sami and non-Sami women, respectively (*p* < 0.001) and a median of 1.33 for both ethnicities (*p* < 0.001) (Table IV). On examining the combined IPV/CV variables, participants that reported IPV or CV had higher mean and median psychological distress and PTS scores compared to the group reporting no IPV and no CV (Tables III and IV). This was observed for emotional, physical, sexual and any IPV, with similar results for Sami and non-Sami women. Those who reported both IPV and CV had the highest mean values. The lowest mean was reported by those reporting no IPV and no CV.

**Table III.** Mean and median Hopkins Symptom Checklist score of 10 questions (HSCL-10) score by different types of violence among Sami and non-Sami women, the SAMINOR 2 Questionnaire Survey (*n* = 6003).

	Sami women *n* = 1197					Non-Sami women *n* = 4806				
	*n*	Mean	SD	Median	Q1–Q3	*n*	Mean	SD	Median	Q1–Q3
HSCL-10 score	1197	1.40^a^	0.497	1.20^b^	1.0–1.6	4806	1.37^a^	0.486	1.20^b^	1.0–1.5
Emotional IPV and/or any CV										
No emotional IPV and no CV	737	1.28	0.382	1.10	1.1–1.4	3466	1.28	0.380	1.10	1.1–1.4
Any CV, but no emotional IPV	312	1.54	0.551	1.40	1.1–1.8	885	1.59	0.631	1.40	1.1–1.9
Emotional IPV, but no CV	86	1.57	0.560	1.40	1.1–1.8	302	1.51	0.553	1.40	1.1–1.7
Emotional IPV and any CV	62	1.94	0.697	1.80	1.4–2.4	153	1.81	0.736	1.60	1.2–2.3
Physical IPV and/or any CV										
No physical IPV and no CV	745	1.28	0.389	1.10	1.0–1.4	3540	1.28	0.387	1.10	1.1–1.4
Any CV, but no physical IPV	313	1.54	0.528	1.40	1.1–1.8	935	1.60	0.629	1.40	1.1–1.9
Physical IPV, but no CV	78	1.54	0.546	1.40	1.1–1.8	228	1.48	0.556	1.30	1.1–1.7
Physical IPV and any CV	61	1.93	0.788	1.80	1.2–2.4	103	1.83	0.805	1.60	1.2–2.3
Sexual IPV and/or any CV										
No sexual IPV and no CV	808	1.30	0.404	1.20	1.0–1.4	3706	1.29	0.395	1.20	1.0–1.4
Any CV, but no sexual IPV	364	1.60	0.595	1.40	1.1–1.9	1013	1.61	0.637	1.40	1.1–1.9
Sexual IPV, but no CV	15	1.69	0.680	1.50	1.0–2.1	62	1.54	0.660	1.30	1.0–1.9
Sexual IPV and any CV	10	1.70	0.614	1.80	1.1–2.2	25	2.21	0.943	2.20	1.3–2.8
Any IPV and/or any CV										
No IPV and no CV	704	1.27	0.382	1.10	1.0–1.4	3393	1.28	0.379	1.10	1.0–1.4
Any CV, but no IPV	278	1.52	0.528	1.30	1.1–1.8	848	1.59	0.633	1.40	1.1–1.9
Any IPV, but no CV	119	1.52	0.518	1.40	1.1–1.8	375	1.48	0.536	1.30	1.1–1.7
Any IPV and any CV	87	1.89	0.709	1.80	1.3–2.4	190	1.77	0.717	1.50	1.2–2.2

Q1, first quartile; Q3, third quartile; IPV, intimate partner violence; CV, childhood violence. ^a^*p* = 0.034 comparing means with independent samples *t*-test.

b *p* < 0.001 comparing the distributions non-parametrically with Wilcoxon rank-sum test.

**Table IV.** Mean and median PTS score by different types of violence among Sami and non-Sami women, the SAMINOR 2 Questionnaire Survey (*n* = 6003).

	Sami women *n* = 1197					Non-Sami women *n* = 4806				
	*n*	Mean	SD	Median	Q1–Q3	*n*	Mean	SD	Median	Q1–Q3
	1197	1.61^a^	0.678	1.33^b^	1.0–2.0	4806	1.49^a^	0.639	1.33^b^	1.0–1.7
Emotional IPV and/or any CV										
No emotional IPV and no CV	737	1.45	0.698	1.30	1.0–1.7	3466	1.37	0.541	1.00	1.0–1.7
Any CV, but no emotional IPV	312	1.79	0.703	1.70	1.3–2.3	885	1.78	0.747	1.70	1.0–2.3
Emotional IPV, but no CV	86	1.81	0.698	1.70	1.3–2.3	302	1.77	0.716	1.70	1.0–2.3
Emotional IPV and any CV	62	2.24	0.872	2.00	1.3–2.8	153	2.04	0.849	2.00	1.3–2.7
Physical IPV and/or any CV										
No physical IPV and no CV	745	1.46	0.589	1.30	1.0–1.7	3540	1.34	0.549	1.00	1.0–1.7
Any CV, but no physical IPV	313	1.80	0.712	1.70	1.3–2.3	935	1.78	0.747	1.70	1.0–2.3
Physical IPV, but no CV	78	1.77	0.713	1.70	1.3–2.3	228	1.75	0.719	1.70	1.0–2.0
Physical IPV and any CV	61	2.19	0.868	2.00	1.3–2.7	103	2.13	0.887	2.00	1.3–3.0
Sexual IPV and/or any CV										
No sexual IPV and no CV	808	1.48	0.604	1.30	1.0–1.7	3706	1.40	0.562	1.00	1.0–1.7
Any CV, but no sexual IPV	364	1.85	0.748	1.70	1.3–2.3	1013	1.80	0.762	1.70	1.3–2.3
Sexual IPV, but no CV	15	1.91	0.728	1.70	1.3–2.7	62	1.77	0.731	1.70	1.0–2.3
Sexual IPV and any CV	10	2.26	0.813	2.30	1.6–2.8	25	2.28	0.874	2.30	1.7–3.0
Any IPV and/or any CV										
No IPV and no CV	704	1.45	0.583	1.30	1.0–1.7	3393	1.37	0.539	1.00	1.0–1.7
Any CV, but no IPV	278	1.77	0.696	1.70	1.3–1.7	848	1.78	0.749	1.70	1.0–2.3
Any IPV, but no CV	119	1.73	0.694	1.70	1.3–2.3	375	1.73	0.701	1.70	1.0–2.0
Any IPV and any CV	87	2.16	0.851	2.00	1.3–2.7	190	1.99	0.825	2.00	1.3–2.7

PTS, post-traumatic stress; Q1, first quartile; Q3, third quartile; IPV, intimate partner violence; CV, childhood violence.

a Comparing means with independent samples *t*-test, *p* < 0.001.

b Comparing the distributions non-parametrically with Wilcoxon rank-sum test, *p* < 0.001.

There were no significant interactions between ethnicity and the combined IPV/CV variables on either psychological distress score or PTS score. Hence, results from the regression analyses for all women combined are presented in Tables V and VI. When adjusting for age in model 1, all types of IPV were strongly associated with psychological distress and PTS scores compared to no IPV and no CV, with the highest scores observed for those reporting both IPV and CV (Tables V and VI). In model 2, adjusting for five additional possible confounders (Laestadian affiliation, municipality of residence, exposure to discrimination, alcohol intake, age and duration of education), the strength of the relationships was reduced but remained statistically significant (Tables V and VI).

**Table V.** Association between Hopkins Symptom Checklist score of 10 questions (HSCL-10) score and intimate partner violence (IPV) and childhood violence (CV) among women, the SAMINOR 2 Questionnaire Survey.

All women (*n* = 6003)	Model 1^a^ (*n* = 6003)		Model 2^b^ (*n* = 5836)	
Predictors	B	*p*-value	B	*p*-value
Emotional IPV and/or any CV				
No emotional IPV and no CV	0		0	
Any CV, but no emotional IPV	0.283	< 0.001	0.244	< 0.001
Emotional IPV, but no CV	0.244	< 0.001	0.213	< 0.001
Emotional IPV and any CV	0.552	< 0.001	0.462	< 0.001
Physical IPV and/or any CV				
No physical IPV and no CV	0		0	
Any CV, but no physical IPV	0.283	< 0.001	0.241	< 0.001
Physical IPV, but no CV	0.221	< 0.001	0.183	< 0.001
Physical IPV and any CV	0.575	< 0.001	0.498	< 0.001
Sexual IPV and/or any CV				
No sexual IPV and no CV	0		0	
Any CV, but no sexual IPV	0.296	< 0.001	0.252	< 0.001
Sexual IPV, but no CV	0.278	< 0.001	0.206	< 0.001
Sexual IPV and any CV	0.755	< 0.001	0.606	< 0.001
Any IPV and/or any CV				
No IPV and no CV	0		0	
Any CV, but no IPV	0.280	< 0.001	0.243	< 0.001
Any IPV, but no CV	0.218	< 0.001	0.189	< 0.001
Any IPV and any CV	0.514	< 0.001	0.432	< 0.001

B, linear regression coefficient.

a Adjusted for age.

b Adjusted for age, duration of education, Laestadian affiliation, municipality of residence, exposure to discrimination and alcohol intake.

Mean score for no emotional IPV and no CV, no physical IPV and no CV, 1.28; no sexual IPV and no CV, 1.29; no any IPV and no CV, 1.37.

**Table VI.** The association between post-traumatic stress (PTS) score and intimate partner violence (IPV) and childhood violence (CV) among women, the SAMINOR 2 Questionnaire Survey.

All women (*n* = 6003)	Model 1^a^ (*n* = 6003)		Model 2^b^ (*n* = 5836)	
Predictors	B	*p*-value	B	*p*-value
Emotional IPV and/or any CV				
No emotional IPV and no CV	0		0	
Any CV, but no emotional IPV	0.379	< 0.001	0.324	< 0.001
Emotional IPV, but no CV	0.392	< 0.001	0.354	< 0.001
Emotional IPV and any CV	0.698	< 0.001	0.598	< 0.001
Physical IPV and/or any CV				
No physical IPV and no CV	0		0	
Any CV, but no physical IPV	0.375	< 0.001	0.318	< 0.001
Physical IPV, but no CV	0.369	< 0.001	0.319	< 0.001
Physical IPV and any CV	0.745	< 0.001	0.643	< 0.001
Sexual IPV and/or any CV				
No sexual IPV and no CV	0		0	
Any CV, but no sexual IPV	0.389	< 0.001	0.329	< 0.001
Sexual IPV, but no CV	0.395	< 0.001	0.313	< 0.001
Sexual IPV and any CV	0.842	< 0.001	0.662	< 0.001
Any IPV and/or any CV				
No IPV and no CV	0		0	
Any CV, but no IPV	0.378	< 0.001	0.325	< 0.001
Any IPV, but no CV	0.345	< 0.001	0.307	< 0.001
Any IPV and any CV	0.647	< 0.001	0.549	< 0.001

B, linear regression coefficient.

a Adjusted for age.

b Adjusted for age, duration of education, Laestadian affiliation, municipality of residence, exposure to discrimination and alcohol intake.

Mean score for no emotional IPV and no CV, 1.39; no physical IPV and no CV, 1.40; no sexual IPV and no CV, 1.41; no any IPV and no CV, 1.39.

When investigating the association between the combined IPV/CV variables and mental health among men, the analyses were performed among all men and for any IPV only, due to small numbers and a lack of significant interaction between ethnicity and the combined variable of any IPV and any CV on psychological distress score or on PTS score. Age-adjusted analyses demonstrated statistically significantly higher mean psychological distress scores in men who reported no IPV and any CV (score 0.24 higher), any IPV but no CV (score 0.26 higher), or any IPV and any CV (score 0.66 higher) than in men who had not experienced IPV or CV (all *p*-values < 0.001) (results not shown in tables). Higher mean PTS scores were also found in men who reported no IPV and any CV (score 0.50 higher), any IPV but no CV (score 0.40 higher), or any IPV and any CV (score 0.80 higher) than in men who had not experienced IPV or CV (all *p*-values < 0.001) (results not shown in tables).

Sensitivity analyses using only participants who reported no violent experiences in their lifetime as the reference group and analyses using the alternative ethnic classification did not render results that changed the conclusions.

## Discussion

The present study uncovered a higher prevalence of IPV among Sami women compared to non-Sami women and among all women compared to all men. IPV was more commonly reported among those who reported CV compared to those who did not report CV. The study demonstrates that emotional, physical and sexual IPV are all associated with mental health problems. The most severe problems were observed among those who reported both IPV and CV. There were no ethnic differences in the associations between the different types of IPV and mental health problems, and we observed similar overall results for men and women. The prevalence of IPV and how IPV, alone or in combination with CV, relates to mental health problems have not previously been studied in a Sami context. The study also presents findings on IPV among men, which is an important contribution to the literature on IPV, as most studies are conducted among only women.

The prevalence of IPV differs between and within countries, and most studies have been conducted among only women [27]. The first national study on IPV in Norway was conducted from 2003–2004 and found a lifetime prevalence of 26.8% among women [28]. This is far higher than the prevalence found in this study (12.8%). This difference in prevalence may be partly explained by the fact that the national study was performed among women who had ever been with a partner, while we did not have information on marital status in the SAMINOR 2 Questionnaire Survey. Another national study in Norway in 2013 found that women reported to have experienced more violent episodes and a higher prevalence of physical IPV than men did (9.2% among women and 1.9% among men). For sexual violence, the numbers were 5.5 and 0.5%, respectively (other types of sexual violence except rape). Rape carried out by a current or former intimate partner was reported by 3.8% of women and 0.1% of men [13]. In comparison, our study found a prevalence of 7.8% for physical IPV in women and 0.4% in men. The prevalence of sexual IPV in our study was lower (1.9% for women and 0.0% for men) than that reported in the national study. The national study did not investigate emotional IPV. The above comparisons suggest that the prevalence figures in our study may be conservative and not inflated. Whether the differences in prevalence are explained by changes over time, regional differences, age differences, or design aspects, is difficult to assess.

In light of previous findings (based on the same sample) of a higher prevalence of violence in general among Sami compared to non-Sami and among all women compared to all men, our results of a similar pattern regarding IPV were probably as expected. A higher prevalence of IPV among indigenous populations compared to the dominant group in their countries has been demonstrated in international studies [16,29,30]. The present results of a higher prevalence of IPV among Sami women are, therefore, in line with those of other studies comparing indigenous populations with the majority population in their countries.

Several researchers have argued that the differences in the prevalence of IPV and mental health problems among indigenous and non-indigenous populations are due to the profound cultural consequences of colonisation, which have left some indigenous populations with poorer social conditions and poor access to healthcare services [17,31]. However, research has shown that, in terms of health, the Sami are better off compared to other indigenous populations [31,32]. Our study found that the mean duration of education was higher for Sami compared to non-Sami women, while no difference was observed between Sami and non-Sami men. In Norway, the educational and healthcare systems are, for the most part, free of charge for all Norwegian residents. This might explain the relatively small ethnic differences in socioeconomic status in Norway as well as the low prevalence of and the relatively small differences in IPV between Sami and non-Sami found in this study.

The fact that we observed similar results in Sami and non-Sami women with respect to the associations between IPV and/or CV and psychological distress and symptoms of PTS, strengthens the assumption that violent victimisation generally affects women`s mental health regardless of ethnic background [7,27,33,34]. The overall results for men resembled those for women, which highlights that, regardless of gender, IPV and CV affect mental health negatively. Further, the findings of higher psychological distress and PTS scores among those who reported both IPV and CV confirm previous findings that exposure to both CV and later, IPV seems to have a strong association with mental health problems [10,35]. Since respondents exposed to violence could indicate their relationship with the perpetrator using alternatives other than the intimate partner (Stranger, Family/relative, or Other acquaintance), the reference group contained both respondents with no violent experiences and respondents exposed to violence from someone other than an intimate partner, which probably attenuated the relationships. Sensitivity analyses were therefore performed using only participants who reported no violent experiences in their lifetime as the reference group, and the results did not change the conclusions. Early life experiences of violence have been cited as risk factors for IPV [10,11]. This is in line with our results, which showed huge differences in the proportion of participants reporting IPV among those exposed to CV compared to those not exposed to CV. In order to prevent this inter-generational cycle of violence, interventions are needed to address CV.

## Strengths and limitations

The study population is a diverse population; all inhabitants, with diverse ethnic backgrounds and of both genders, in preselected municipalities with a high proportion of Sami were invited to participate. This study had a cross-sectional design that limited the potential to assess a causal link between the different types of violence and mental health problems. However, exposure to CV and IPV is likely to have taken place prior to the reported mental health problems.

The HSCL-10 is widely considered a reliable and valid instrument to measure psychological distress [25]. A study that used the same dataset as our study, found no significant measurement invariance between Sami and non-Sami, indicating that Sami and non-Sami interpret the HSCL-10 items in a similar way [36].This is a strength of the study. The mean HSCL-10 score in our study among non-Sami respondents is consistent with the mean value of the HSCL-25 in a national study [37]. This suggests that our estimates are valid. However, only three questions from the NorAQ were used to assess symptoms of PTS. Although these questions cover core symptoms included in the Diagnostic and Statistical Manual of Mental Disorders (DSM-V), they are not sufficient to meet all the DSM-V criteria for a PTSD diagnosis. A major limitation is that the PTS questions were not asked in response to a specific stressor. Hence, we do not know whether the reported exposure is a traumatic event according to the criteria in the DSM-V for a PTSD diagnosis. Despite this major limitation, the internal consistency of these items was acceptable (Cronbach’s alpha 0.75) for both ethnicities, strengthening the reliability of the measurements. However, more items measuring symptoms of PTS would strengthen the validity of the instrument. The three NorAQ questions that we used were used in other studies [34,38]. The questions used to assess violence were also taken from the NorAQ. A validation study among women and men showed that the NorAQ had good validity and reliability [39]. However, the questions used in this study represent a modified version of the NorAQ, and they have not been validated in the Sami population or in the non-Sami population in rural Northern Norway. Thus, differences in cultural and linguistic interpretations may have influenced the observed differences between the two groups. However, the questions were formulated rather broadly, which may reduce potential bias based on cultural differences. Potential misclassification in the Sami group is unlikely, as studies have found Sami self-identification to be relatively stable [40]. However, due to harsh assimilation policies, many Sami have abandoned or denied their Sami ethnicity. A potential misclassification of Sami into the non-Sami group might therefore have occurred, and the association between ethnicity and mental health problems may have been attenuated. There is no official register of Sami ethnicity in Norway and no consensus on how to define Sami ethnicity. Other studies have used different criteria to define the Sami groups. Therefore, we conducted additional analyses using an alternative ethnic categorisation in which we included the criteria ‘My ethnic background is Sami’ to our definition of Sami ethnicity. This definition has been used in several papers [23,41,42]. Further, we conducted additional analyses with a reference group that reported no violent experiences in their lifetime. The strength of the association with mental health problems did not considerably change in these sensitivity analyses, and this is a strength of the study.

Due to the low participation rate in the SAMINOR 2 Questionnaire Survey, selection bias is likely, and the prevalence estimates must be interpreted with caution. We have limited information about non-respondents, namely that participation increased with age, and more women than men participated [22]. It is possible that differences in response rates between Sami and non-Sami may have influenced the prevalence of IPV and CV in each group. As ethnicity is not recorded in any official register in Norway, we were not able to assess whether the proportion of non-respondents differed in Sami and non-Sami. However, a comparison has been made between respondents and non-respondents of the SAMINOR 2 Questionnaire Survey among invitees who had previously participated in the SAMINOR 1 Survey from 2003–2004 [22]. In this selected group, questionnaire information from the SAMINOR 1 Survey was used to compare respondents and non-respondents of the SAMINOR 2 Questionnaire Survey. The proportions reporting Sami affiliation in SAMINOR 1 were similar among respondents and non-respondents, indicating that response rates did not differ by ethnicity. It was found that, compared to the non-respondents, the respondents had higher education levels and family incomes.

A general tendency to underreport taboo topics like CV and IPV [37] may have introduced bias, thus diminishing the association with adult mental health problems. Conversely, current anxiety and depression may increase the tendency to recollect and report life events in a more negative and traumatic way [43], thus strengthening the association with mental health problems. Recall bias on the outcome variables is considered to be of minor importance, as the respondents were asked about recent mental health.

Due to the low response rate, cross-sectional design and potential biases, our results must be interpreted with caution. However, we regard the finding of an association between IPV/CV and mental health problems to be valid based on the very strong associations observed and the consistency of results across gender and Sami/non-Sami ethnicity. Further research is needed to confirm our results.

## Conclusion

The present findings demonstrate that exposure to emotional, physical, or sexual IPV is strongly associated with mental health problems. The most severe mental health problems were observed for those who were exposed to both IPV and CV. It is therefore important for victims of IPV to address experiences of violence that occurred earlier in life. The strength of the association between the different types of IPV (emotional, physical and sexual) and mental health problems was quite similar. Results for Sami and non-Sami women did not differ, and overall we observed the same pattern for men as well. A higher proportion of Sami women reported emotional and physical IPV compared to non-Sami women, while there were no ethnic differences in sexual IPV. Women in general reported a higher prevalence of emotional, physical, and sexual IPV than men. Although there may be gender and ethnic differences in the exposure to IPV and CV, the association between exposure to IPV and CV and mental health seems to be the same, regardless of ethnicity and gender.
